# Treatment of Gingival Hyperpigmentation by Diode Laser for Esthetical Purposes

**DOI:** 10.3889/oamjms.2015.071

**Published:** 2015-08-07

**Authors:** Hanaa M. El Shenawy, Sherine A. Nasry, Ahmed A. Zaky, Mohamed A. A. Quriba

**Affiliations:** 1 *Orodental Division Department, National Research Centre, Cairo, Egypt*; 2 *Medical Laser Application Department, National Institute of Laser Enhanced Science (NILES), Cairo University, Cairo, Egypt*

**Keywords:** diode laser, gingival, hyperpigmentation, melanin

## Abstract

**BACKGROUND:**

Gingival hyperpigmentation is a common esthetical concern in patients with gummy smile or excessive gingival display. Laser ablation has been recognized recently as the most effective, pleasant and reliable technique. It has the advantage of easy handling, short treatment time, hemostasis, decontamination, and sterilization effect.

**AIM:**

In the present study we wanted to explore the efficacy of a 980 nm wavelength diode laser in gingival depigmentation clinically by using both VAS and digital imaging method as means of assessment.

**METHODS:**

Diode laser ablation was done for 15 patients who requested cosmetic therapy for melanin pigmented gums. The laser beam delivered by fiberoptic with a diameter of 320 µm, the diode laser system has 980 nm wave lengths and 3 W irradiation powers, in a continuous contact mode in all cases, the entire surface of each pigmented maxillary and mandibular gingiva that required treatment was irradiated in a single session. Clinical examination and digital image analysis were done and the patients were followed up for 3 successive months.

**RESULTS:**

There was a statistically significant change in prevalence of bleeding after treatment, as none of the cases showed any signs of bleeding 1 week, 1 month and 3 months after ablation. No statistically significant change was observed in the prevalence of swelling after treatment The VAS evaluation demonstrated that only 4 patients complained of mild pain immediately after the procedure. No pain was perceived from the patients in the rest of the follow up period. There was no statistically significant change in prevalence of pain immediately after treatment compared to pain during treatment. There was a decrease in cases with mild pain after 1 week, 1 month as well as 3 months compared to pain during treatment and immediately after treatment.

**CONCLUSION:**

Within the limitations of this study, the use of diode laser was shown to be a safe and effective treatment modality that provides optimal aesthetics with minimal discomfort in patients with gingival hyperpigmentation.

## Introduction

Gingival hyperpigmentation is increased pigmentation beyond the normally expected degree of the oral mucosa. Several physiologic and/or pathologic factors can cause hyperpigmentation [1]. However the most common cause is physiologic or ethnic hyperpigmentation. Physiologic hyperpigmentation is genetically determined and is clinically manifested as variable amounts of diffuse or multifocal melanin pigmentation in different ethnic groups [2].

Melanin, a brown pigment, is the most common natural pigment contributing to endogenous pigmentation of gingiva and is produced by melanocytes in the basal and supra-basal cell layer of the gingival epithelium [3]. The gingiva is the most frequently pigmented tissue of the oral cavity [4].

Although gingival melanin pigmentation does not represent a pathological problem, patients with a gummy smile or excessive gingival display usually complain of a “black gum” and request cosmetic therapy [5, 6].

Gingival depigmentation is a treatment to remove melanin hyperpigmentation of the gingiva and various methods have been used for this procedure with different degrees of success including gingivectomy [7], gingivectomy with free gingival autografting [8], electrosurgery [9], Cryosurgery [10], chemotherapy with 90% phenol and 95% alcohol and abrasion with diamond bur [11]. Moreover some of these techniques are prone to side effects and complications [12]. Recently lasers have been used to ablate cells containing and producing the melanin pigment [5]. The commonly used lasers for gingival de-epithelization include semi-conductor diode, Er: YAG Nd: YAG laser, and CO2 laser

Recent research has centered on using pulsed diode laser (810 λ) for oral surgery of the tongue and gingiva and to remove infected epithelium in chronic periodontitis. The advantages of this laser in its easy gingival reshaping, reduced need for local anesthesia, excellent hemostasis, minimal thermal injury of the deeper tissues and negligible post-operative pain and inflammation [12, 13]. Moreover there is evidence in the recent literature of successful depigmentation using diode lasers [2].

Digital cameras produce color images that consist of three – red (R), green (G) and blue (B) – components, and the corresponding three spectrally-selective images can be reconstructed and analyzed additionally to the conventional color image [14-16]. RGB camera works as a simple multi-spectral imaging device acquiring three spectral images at a time where R channel can be roughly attributed to 600…700 nm spectral range, G – 500…600 nm and B – 400…500 nm. Data fitting and estimation techniques are then utilized to obtain estimates of hemoglobin and melanin distribution [16]. Advantage of such simplified approach is a possibility to acquire the spectral image cube immediately, without time losses due to durable signal processing [14-16].

In the present study we wanted to explore the efficacy of a 980 nm wavelength diode laser in gingival depigmentation clinically by using both VAS and digital imaging method as means of assessment. Long-term monitoring and direct visual comparison of medical states of oral pathologies on the basis of individual subjects can be applied. Even though colorimetric values of the pixels in the images are in device dependent RGB color space of the camera, certain analyses of color properties in images acquired with the proposed acquisition method are possible. One such application could be to monitor changes in gingival or teeth colors on the basis of individual subjects over a long period of time.

## Material and Methods

Fifteen patients (7 males and 8 females) suffering from gingival hyper pigmentation in the anterior segment of the mouth with age range from 15 to 45 years old, and free from any systematic diseases which may have effect on healing post operatively, was included in this study. Patients were selected randomly from the outpatient clinic of the National Institute of Laser Enhanced Science (NILES), Cairo University, Egypt and assessed for eligibility by an oral Medicine, Diagnosis and Periodontology specialist. The patients consented to their enrollment in the study by signing a written informed consent. Patients were aware of the nature of ethnic/physiological hyperpigmentation and understood that this phenomenon had no influence on their systemic or oral health.

Inclusion criteria was moderate to severe bilateral melanin hyperpigmentation of the upper and lower gingivae as given by Dummett Gupta in 1964 [17] and well maintained oral hygiene and esthetic concerns. Exclusion criteria were: history of systemic diseases associated with pathological hyperpigmentation or improper delayed wound healing (uncontrolled diabetes, autoimmune diseases, etc.), pregnancy and lactation, untreated periodontal disease, chronic smokers and non-compliant patients [18].

### Clinical examination

Clinical Assessment of swelling and bleeding was done immediately after termination of laser ablation, after 1week and 1 month and 3 months postoperatively. The VAS [19] was used to measure the intensity of pain experienced during and after treatment. The VAS consisted of a horizontal line 100 mm long, anchored at the left end by the descriptor ‘‘no pain’’ and at the right end by ‘‘unbearable pain.’’ The patient placed a mark to coincide with the level of pain. The distance of this point, in millimeters, from the left end of the scale was recorded and used as the VAS score. Scores were calculated as: 0 =no pain; 0.1 to 3.0 cm (1 to 30 mm) = mild pain; 3.1 to 6.0 cm (31 to 60 mm) = moderate pain; 6.1to 10 cm (61 to 100 mm) = severe pain

### Digital examination

The change in gingival pigmentation was also studied using comparative clinical photographs. Four photos were taken for each patient, the first before laser application, then one weak, one month, and 3 months after laser treatment comprising a total of 60 photos for 15 patients.

Image analysis was done by using a high resolution digital camera (Nikon Coolpix 1810). Photos were taken by the same camera and with the same zoom and the same programmed chair position to ensure standardization. The distance between the camera and the patient was also standardized at 3 feet by placing the camera over its tripod stand. The triple feet of the camera stand were then marked on the floor using permanent marker.

The RGB (red, green and blue) value was analyzed using Adobe Photoshop CS5 version to analyze the histogram of the digital photographs.

### Laser procedure

The diode laser used in this study has fiberoptic delivery system with beam diameter of 320 µm, 980 nm wave length and was operated at a 3 W irradiation power, in a continuos contact mode.

Before applying the laser, the operating staff and the patients wore special laser-protective eye glasses corresponding to laser wavelength. Highly reflective instruments or instruments with mirrored surfaces were avoided [20].

In all cases, the entire surface of each maxillary and mandibular gingiva that required treatment was irradiated in a single session.

Precautionary postoperative instructions such as avoiding smoking and eating hot and spicy food for the first 24 hours were given to all patients. Also patients were advised not to traumatize the area during the healing period which is 4 -7 days after treatment and to take analgesics ibuprofen 200 mg after the surgery and to continue with the medication for the next 3 days if pain was experienced.

After application of topical anesthesia (lignocaine hydrochloride), laser ablation was started from the mucogingival junction working toward the free gingival margin, including the papillae in a continuous contact mode with overlapping circles and the fiber tip was continuously moved across the site to avoid heat accumulation at any site. The water spray helped to rinse the tissue as ablation proceeded. Then the area was wiped with gauze soaked in normal saline. The same procedure was repeated till no pigments remained.

No periodontal dressing was placed and no antibiotics were prescribed. The procedure was completed within 20 to 25 minutes. The patients were then followed up for 3 subsequent visits, after one week, one month then 3 months.

### Statistical analysis

Qualitative data were presented as frequencies (n) and percentages (%). Cochrane’s Q and Friedman’s tests were used to study the changes after treatment in different qualitative data. Age data were presented as mean, standard deviation (SD), minimum and maximum values. The significance level was set at P ≤ 0.05. Statistical analysis was performed with IBM® SPSS® Statistics Version 20 for Windows.

Data collected from the pixel profile program was collected, tabulated and analyzed using SPSS V.16 and Gragh PAD Prisn V.6.

### Clinical Results

The 980 nm diode laser used with water spry effectively ablated the epithelial tissue exhibiting melanin pigmentation. Most of the melanin pigmentation was removed with minimal penetration into the tissue. The procedure time ranged from 20 to 25 min depending on the severity and extent of pigmentation. In all patients immediately after the procedure, gingival connective tissue was exposed with slight bleeding. The treated area appeared white due to the formation of protein coagulum and there was no need to apply a periodontal dressing. The treated surface did not exhibit major thermal changes, such as marked coagulation and carbonization. A white fibrin slough was seen after 24 hours. At 1 week, the treated gingiva showed fast epithelization with a healthy appearance, but immature healing, in all cases (Fig. 2).

**Figure 1:**
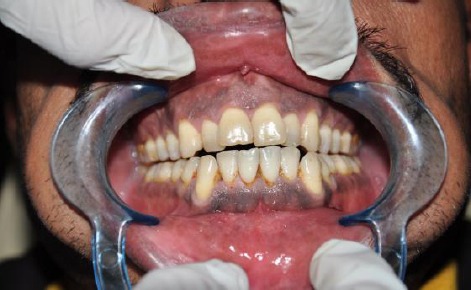
*Preoperative view showing diffuse melanin pigmentation*.

**Figure 2:**
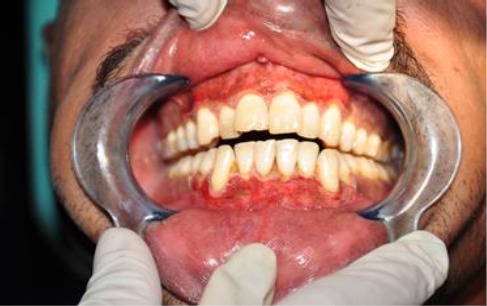
*Post operative view 1 week later*.

At the second week the epithelium showed a non-keratinized translucent appearance and the laser-irradiated gingiva appeared reddish compared to the neighboring untreated gingiva. At 1 month, complete healing with tissue maturation was observed and the gingiva exhibited normal appearance (Fig. 3). Postoperative side effects such as gingival recession were not observed in any of the cases during the 3 months observation period.

**Figure 3:**
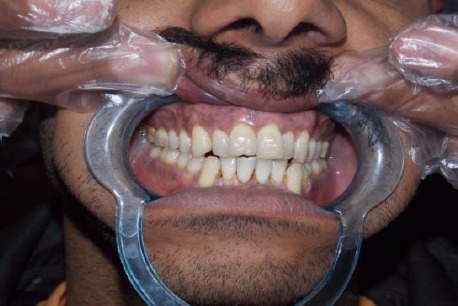
*Postoperative view one month later*.

**Figure 4:**
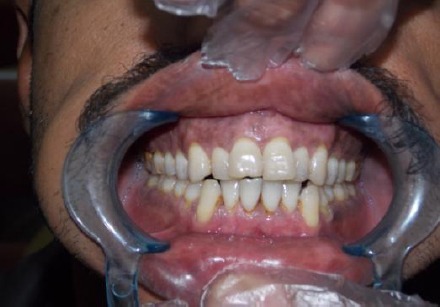
*Postoperative view three month later*.

The treated sites showed uneventful wound healing without any severe post-surgical problems. There was obvious change in the amount of the pigmented areas in the patients gingiva before and immediately after the operation and during the post-operative visits (Fig. 1-4). Also the digital analysis of the photographs by using Photoshop software showed a decrease in the pigmentation by RGB factor. All of the melanin pigmentation was removed with minimal penetration into the tissue. There were no areas of carbonization and the bleeding was controlled. During the three months follow up no signs of re-pigmentation were observed.

The achieved results were satisfactory for patients and the operator and met the patient’s expectations.

### Statistical analysis

Qualitative data were presented as frequencies (n) and percentages (%). Cochrane’s Q and Friedman’s tests were used to study the changes after treatment in different qualitative data.

Numerical data were presented as mean and standard deviation (SD) values. Data were explored for normality using Kolmogorov-Smirnov and Shapiro-Wilk tests. All data showed parametric distribution except for (Y) and (H) values.

For parametric data, repeated measures ANOVA test was used to study the changes by time. Bonferroni’s post-hoc test was used for pair-wise comparisons when ANOVA test is significant. For non-parametric data, Friedman’s test was used to study the changes by time. Wilcoxon signed-rank test was used for pair-wise comparison when Friedman’s test is significant. Bonferroni’s adjustment was applied for the pair-wise comparisons.

The significance level was set at P ≤ 0.05. Statistical analysis was performed with IBM® SPSS® Statistics Version 20 for Windows.

## Results

### Base line characteristics

The present study was conducted on 15 patients; 7 males (46.7%) and 8 females (53.3%). The mean ± standard deviation (SD) values of age were 29.9 ± 7.0 years with a minimum of 18.0 years and a maximum of 40.0 years old. Twelve cases (80%) had moderate pigmentation while three cases (20 %) had severe pigmentation.

There was slight bleeding in only 3 cases immediately after treatment, while the remaining cases showed no bleeding signs. There was a statistically significant change in prevalence of bleeding after treatment, as none of the cases showed any signs of bleeding 1 week, 1 month and 3 months after ablation (Table 1, Fig. 1).

**Table 1:** Frequencies (n), percentages (%) and results of Cochrane’s Q test for the comparison between bleeding before and after treatment

*Bleeding*	*Immediate after treatment*		*1 week*		*1 month*		*3 months*		*P*-value

	*n*	*%*	*n*	*%*	*n*	*%*	*n*	*%*	
No bleeding	12	80.0	15	100.0	15	100.0	15	100.0	0.029*
Slight bleeding	3	20.0	0	0.0	0	0.0	0	0.0	

* Significant at P ≤ 0.05

**Figure 1:**
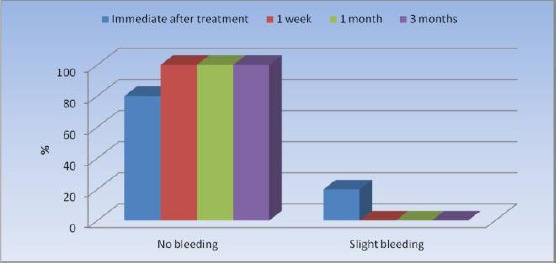
*Bar chart representing bleeding before and after treatment*.

No statistically significant change was observed in the prevalence of swelling after treatment as only two cases displayed signs of swelling immediately after treatment (Table 2, Fig. 2).

**Table 2:** Frequencies (n), percentages (%) and results of Cochrane’s Q test for the comparison between swelling before and after treatment

*Swelling*	*Immediate after treatment*		*1 week*		*1 month*		*3 months*		*P*-value

	*n*	*%*	*n*	*%*	*n*	*%*	*n*	*%*	
No swelling	13	86.7	15	100.0	15	100.0	15	100.0	0.112
Slight swelling	2	13.3	0	0.0	0	0.0	0	0.0	

* Significant at P ≤ 0.05

**Figure 2:**
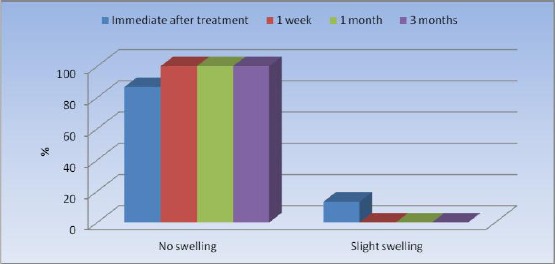
*Bar chart representing swelling before and after treatment*.

From the VAS evaluation only 4 patients complained of mild pain immediately after the procedure. No pain was perceived from the patients in the rest of the follow up period. There was no statistically significant change in prevalence of pain immediately after treatment compared to pain during treatment. There was a significant decrease in cases with mild pain after 1 week, 1 month as well as 3 months compared to pain during treatment and immediately after treatment (P= 0.08) (Table 3, Fig. 3).

**Table 3:** Frequencies (n), percentages (%) and results of Cochrane’s Q test for the comparison between pain before and after treatment

*Pain*	*During treatment*		*Immediate after treatment*		*1 week*		*1 month*		*3 months*		*P*-value

	*N*	*%*	*N*	*%*	*n*	*%*	*n*	*%*	*n*	*%*	
No pain	11	73.3	12	80.0	15	100.0	15	100.0	15	100.0	0.008*
Mild pain	4	26.7	3	20.0	0	0.0	0	0.0	0	0.0	

* Significant at P ≤ 0.05

**Figure 3:**
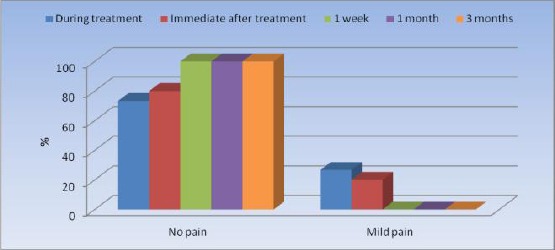
*Bar chart representing pain before and after treatment*.

### Laser parameters (R, G, B)

To study the change in gingival color, a total of 60 photos where included for image analysis with the pixel profile program to detect the change of the RGB of the image before treatment 1 week, 1 month and 3 months post treatment for each patient.

Data collected from the pixel profile program were tabulated and analyzed using SPSS V.16 and Gragh PAD Prisn V.6 then Photoshop CS5 was used to cut the photos. A multiple comparison test was done to test the relation between visits.

Concerning the component R, there was no statistically significant difference between the four visits in the change of the red color for haemoglobin (P = 0.156), while, there was statistically significant difference between the four visits in the green color for cytoplasm (P = 0.012), and a highly statistically significant difference was found between the four visits regarding the blue color for melanin component B (P = 0.000).

## Discussion

Melanin pigmentation of the gingiva may be seen across all races, at any age and without gender predilection. Although it does not present a medical problem, complaints of black gum and a demand for depigmentation is common. Many techniques have been tried for depigmentation.

**Table 5:** Descriptive Analysis for Hyperpigmentation PIXELES analysis

Color Elements	Visits	Pixel (Mean ± SD)	P value
**R (red)**	Pre treatment	175.8 ± 21.52		

	Week later	201.61 ± 26.58	Ns	

	Month later	184.9 ± 36.45		

	3 Months later	184.67 ± 36.83		

**G (green)**	Pre treatment	125.34 ± 23.05		

	Week later	98.48 ± 24.83	**	

	Month later	107.3 ± 19.74		

	3 Months later	107.12 ± 19.74		

**B (blue)**	Pre treatment	128.2 ± 23.27		

	Week later	83.35 ± 33.26	***	

	Month later	104.2 ± 19.01		

	3 Months later	104.1 ± 18.97		

Ns = non significant

** significant

*** highly significant.

Recently laser ablation has been recognized as one of the most effective, comfortable and reliable techniques for gingival depigmentation [21]. Gingival depigmentation performed in this study was carried out by a 980 nm and a 4 W irradiation power settings diode laser (quanta laser system made in Italy 980 nm class 4 laser) as it has near optimal absorption for melanin and hemoglobin Moreover in comparison with Er: YAG, diode laser offers the advantage of a successful and safe application, being able to prevent bleeding, limit postoperative inflammation and pain and favor healing of gingival mucosa [12].

In the present study minimal side effects such as the very slight coagulation on the treated surface without major thermal side effects such as carbonization and severe coagulation that could interfere with wound healing process was possible with 980 nm diode laser.

The procedure was done in a contact mode allowing good tactile sensation and precision while operating. Clinical results indicating a safe, effective and practical melanin pigmentation ablation have been reported with this contact mode. Using the contact mode with water spray was reported to achieve precise irradiation, good tactile sensation and reduced thermal effect in a clean operating environment [4, 5, 22].

Moreover complete de-epithelization requires that the instruments are used in contact mode, in order to get optimal control of the laser beam without damaging the neighbouring teeth, and alveolar bone [23]. The effectiveness of this method was confirmed in the present study.

The white fibrin slough seen in after 24 hours in all patients is due to the relatively thick coagulation layer on the treated surface produced by the “hot tip” of the diode laser fiberoptic. This is a normal characteristic of a laser wound during the first several days of healing. Mild pain, swelling and bleeding were observed by some patients immediately after surgery, while these inflammatory signs subsided during the whole follow-up periods. The lack of bleeding after laser treatment can be attributed to the property of lasers to coagulate blood vessels and thereby assist in providing a relatively dry surgical field [11]. Laser is absorbed by pigments in the soft tissue, thus making it an excellent hemostatic agent [20]. The slight bleeding observed by some patients after surgery might be due to the laser beam penetrating deeper than required. This is in accordance with Kishore et al 2014 who observed that the bleeding was directly correlated with the depth of ablation [24]. The use of water enhanced the visualization of the operative field and minimized heat generation by cooling the irradiated area and absorbing excessive laser energy [4].

It was theorized that the protein coagulum formed on the wound surface as a result of irradiation might act as a biological wound dressing sealing the ends of sensory nerve endings. In a study comparing the VAS score for patients treated with laser was lower compared to patients treated with scalpel surgery and electrosurgery, indicating that laser procedure produced less pain and discomfort The lack of swelling after laser treatment is in line with Khakhar et al who reported complete removal of the gingival epithelium without causing microvessel dilatation and is possibly related to the direct vasomotor effects and/or deactivation of local pro-inflammatory mediators by the diode laser light causing microvessel narrowing [25]. The mild inflammatory signs manifested as swelling and bleeding observed immediately after surgery by a few patients might be because of the deep pigmentation in these patients, while the pain observed during and immediately after surgery might be attributed to a low threshold of pain in these cases. Most adverse effects and complications of laser treatment can be predicted by understanding that they are mainly due to collateral damage of normal adjacent structures.

Successful treatment following laser ablation of hyper-pigmented areas was evident by the uneventful healing of the gingiva and complete regeneration resulting in a healthy pink firm appearance. These findings confirm and extend the previous data on the successful application of laser techniques for the treatment of gingival hyperpigmentation [5]. Photomodulation effects of laser were shown to help in stimulating the fibroblasts, angiogenesis and accelerating the lymphatic flow, which enhances repair and regeneration. In addition the bactericidal effect of laser related to the generation of reactive oxygen species may also add to the faster healing in a relatively sterile environment [26].

The laser procedure was acceptable to the patients as the procedure took less time and was comfortable because the treated area required no painful injections and patients experienced no potoperative pain and injections after being dismissed. Similar results were reported by other studies who stated that diode laser presented advantages in terms of less discomfort /pain during post-therapy period and a reduction of treatment chair time [27, 28].

Digital color imaging acquisition is commonly comprised of three broadband filtered images (red, green, blue) approximating the light sensitivity of the cones in the human eye. The blue color channel is the component of the RGB color system that best represents the dark shade of melanin, the red represents the heamoglobin and the green represents the cytoplasm. This means that the pixels of the image can be interpreted as shinning points with intensities of color that can be decomposed into the RGB channels. The index values derived from digital images depends on the camera used as well as on the circumstantial conditions, such as the distance from objects and illumination [29].

Digital image analysis is commonly applied for studying skin lesions [14, 29, 30], however to our knowledge this is the first study to use this type of analysis on intraoral mucosal lesions.

In the present study photos were taken by the same camera and with the same zoom and the same programmed chair position to ensure standardization. The distance between the camera and the patient was also standardized at 3 feet by placing the camera over its tripod stand.

Results demonstrated no statistically significant difference between the four visits in the change of the red color, although there was a slight increase in the red component in the first week after ablation denoting the active healing process as granulation tissue appeared red in color due to the pigment hemoglobin content in the blood. In a study on the effect of nanosecond-domain laser pulses on the pigmentary system of the skin, each wavelength, melanosomes were ruptured within keratinocytes and melanocytes, with cytoplasmic and nuclear alterations. Delayed epidermal depigmentation occurred, followed by gradual repigmentation [31]. Statistical anlaysis in the present study revealed significant difference between the four visits in the green color (P =0.012) caused by pixel changes, denoting alterations in the cytoplasmic content.

A highly statistically significant difference was found between the four visits regarding the blue color component, denoting the significant decrease in the amount of pigmentation after laser ablation then the melanin pigment amount became constant during the next follow up sessions indicating that there was no repigmentation during the follow up period.

However, because of the short follow-up period in this study, these results may not be conclusive. In most techniques partial repigmentation appeared in about half of the patients after 2-4 years of treatment [5]. As the success of depigmentation procedure does not only rely on the amount of depigmentation achieved but also by the time taken for repigmentation to appear, and because the postoperative follow-up period of this study was short, further studies with prolonged follow up is advised.

In conclusion, within the limitations of this study, the use of a diode laser was shown to be a safe and effective treatment modality to provide optimal aesthetics with reduced discomfort to the patients during the treatment for gingival hyper pigmentation
